# Retrospective digital study of mandibular flexure in patients with long-span fixed restorations supported by natural teeth

**DOI:** 10.1186/s13104-023-06486-w

**Published:** 2023-09-11

**Authors:** Hussein El Charkawi, Hossam I. Nassar, Medhat Sameh Abdelaziz

**Affiliations:** Department of Prosthodontics, Faculty of Oral and Dental Medicine, Future University in Egypt, Fifth Settlement, End of 90 Street, New Cairo, Egypt

**Keywords:** Long-span fixed prosthesis, Mandibular flexure, Digital analysis

## Abstract

**Purpose:**

This retrospective study aims to evaluate the mandibular flexure on a long-span rigid fixed prosthesis supported by natural teeth.

**Materials and methods:**

Nine patients (five males and four females) were included in this study who had long-span rigid mandibular fixed prostheses for long-term (10–15 years) that have led to radiographic changes around the supporting teeth. The mandibular flexure was measured digitally after adhering reference markers to the prostheses. Intraoral scans were obtained at the minimum and maximum mouth openings before and after splitting the preexisting prostheses. The distances between the markers were measured, and mandibular flexure was calculated.

**Results:**

This study showed a significant deviation (narrowing) of the mandible before and after splitting the rigid fixed prostheses (P value < 0.05).

**Conclusion:**

Digital analysis of the data collected from the patients in this retrospective study indicated that deviations occur during mandibular flexure.

**Clinical relevance:**

Splitting the full arch prosthesis could prevent the negative consequences of mandibular flexure on restorations.

*Trial registration* The study was registered on clinicaltrials.gov with registration number NCT05617274 (15/11/2022)

## Introduction

Medial mandibular flexure is a deformation of the mandible, characterized by a decrease in the width of the arch during maximum jaw opening, due to the functional contraction of the lateral pterygoid muscle. During these movements, the muscle activity makes both mandibular rami approach, leading to a reduction in the intercondylar distance and causing high strain in the symphysial region [1]. Medial mandibular flexure is considered a complex and multifactorial deformation of the mandible, that causes significant changes in the shape of the mandible [2].

The lateral pterygoid muscles on both sides and the muscles of the floor of the mouth exert a force on the mandible. Mandibular flexion was also observed during clenching, occlusion, or biting in the mandible [3–6]. Metal and zirconia fixed prostheses have low elasticity, which greatly limits mandibular flexure, especially when the entire natural dentition is splinted with one unit of a metal-ceramic or zirconia prosthesis [7].

Accordingly, in need of long-span mandibular fixed restorations, segmented bridges are the more predictable practice to counteract the mandibular deformation during wide mouth opening and its potential negative consequences [8, 9]. These consequences ranged from pain at certain sites like the corner of the mouth, severe bone resorption, cystic formation, tooth mobility, porcelain chipping, and sometimes breakage of the prosthesis [7, 10–12]. These negative consequences of bone resorption require the application of bone grafts and pericardium membranes [13, 14].

This study aimed to evaluate the effect of long-span fixed prostheses on mandibular flexure. The null hypothesis tested in this retrospective study was that there will be no significant difference in mandibular flexure before and after splitting the long-span teeth-supported fixed restorations.

## Materials and methods

The patients included in this study were referred for prosthetic consultations over the past 2 years (from June 2020 to August 2022). All had the entire mandibular natural dentition (6–8 abutments) splinted with a single-unit metal-ceramic (long-span fixed prosthesis). All of them suffered from different negative consequences around the natural teeth supporting the prosthesis. The retrospective collection of the patient’s data was tabulated and analyzed. Nine patients were included in this study. Five males and four females with an age range of 50–76 years were enrolled in this study. The sample size was calculated according to an expert opinion due to the limited number of patients that have long span fixed mandibular prosthesis that requires removal also, there was no previous study that measured mandibular flexure in this group of patients. The inclusion criteria were fixed full-arch mandibular single prostheses that have been serviced intraorally for many years (average 10–15 years) and are opposed by natural dentition. All patients were free from any systemic diseases such as bone diseases, diabetes mellitus, or blood dyscrasias. Patients with temporomandibular joint dysfunction, maxillofacial surgeries, or mandibular trauma were excluded from the study.

The patient’s approval to be enrolled in this study and be recalled for follow-up appointments was documented in written and signed consent forms. The investigation was conducted in full accordance with the applicable ethical principles, including those of the World Medical Association and the Declaration of Helsinki. The present study was approved by the Ethics Committee of the Future University in Egypt with reference number (FUE.REC (26)/10-2022). The study was registered on clinicaltrials.gov with registration number NCT05617274. (15/11/2022).

**Fig. 1:**
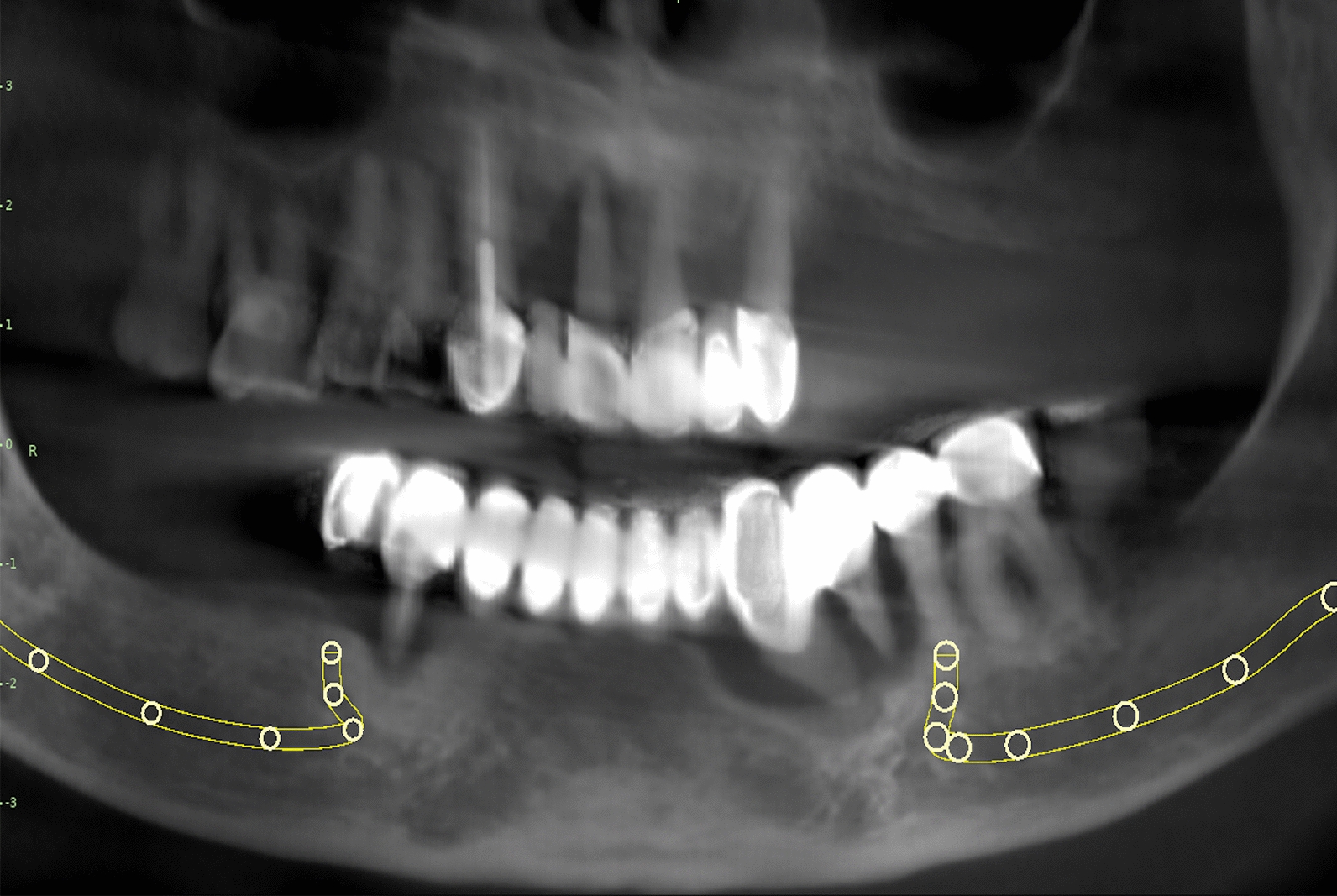
Panoramic X-ray showing bone loss around the natural teeth for a patient having long-span fixed prosthesis

**Fig. 2:**
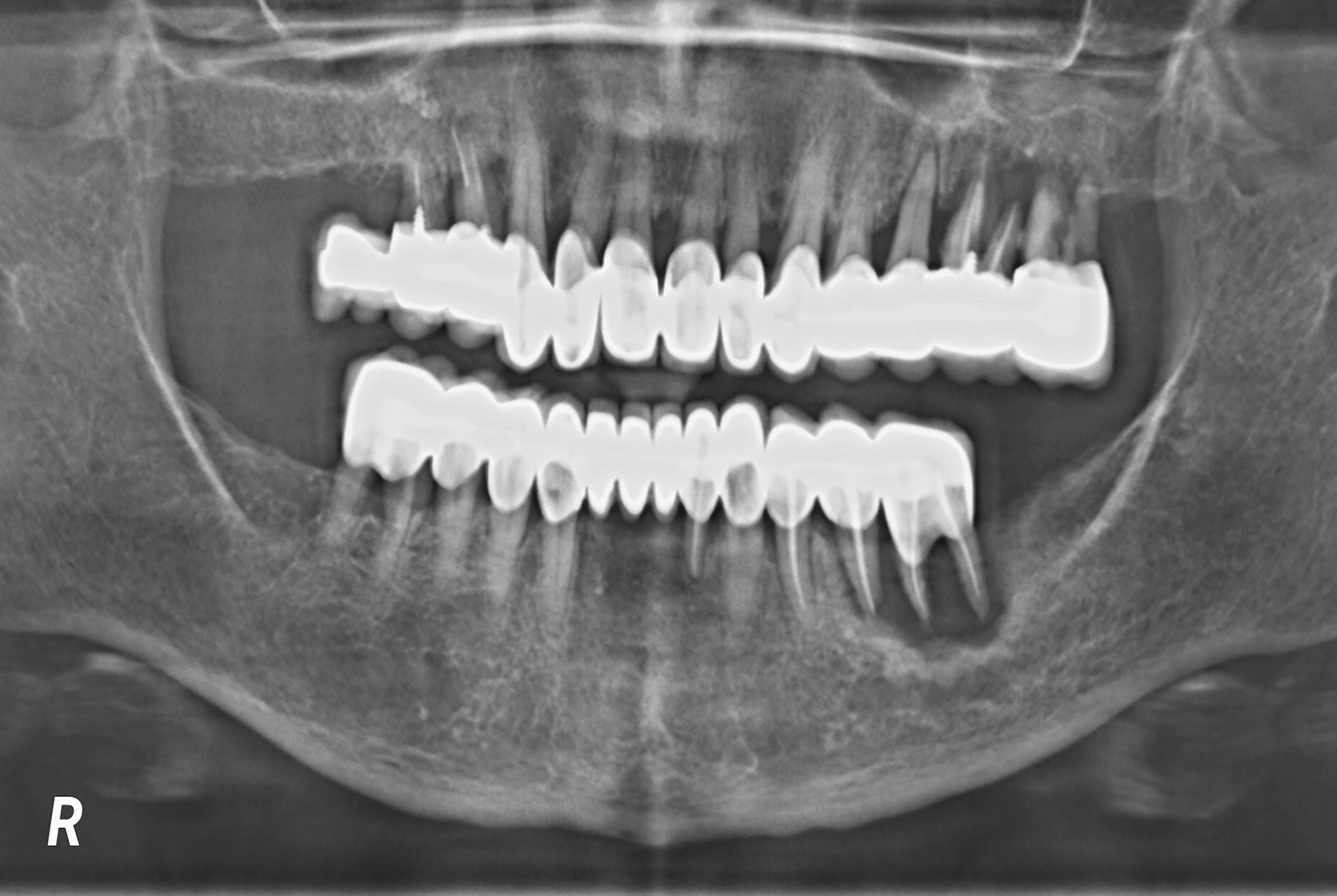
Panoramic X-ray showing bone loss around the posterior teeth for a patient having long-span fixed prosthesis

A panoramic radiograph and Cone Beam Computed Tomography (CBCT) of the mandible were carried out for all patients. The radiographic examination revealed severe bone resorption at premolar-molar sites in all patients, associated with small and medium-sized odontogenic mandibular cysts in most of them, as detected by pathological examination (Figs. 1, 2). The treatment plans were set after a complete analysis of the clinical and radiographic data collected. The treatment plans included: (1) sectioning and removal of the existing prostheses; (2) extraction of the non-restorable supporting teeth; (3) management of the cystic lesions by enucleation and bone augmentation in patients with cystic formation; and (4) restoring the mandible with non-splinted (segmented) implant-supported prostheses (from 6 to 8 implants distributed over the entire residual ridge) [15].

To measure mandibular flexure values, reference aids made of four black endodontic rubber stoppers that were adhered to the surface of the prosthesis at the canine (anterior reference points) and the first molar or second premolar (posterior reference points) on each side of the prosthesis.

**Fig. 3:**
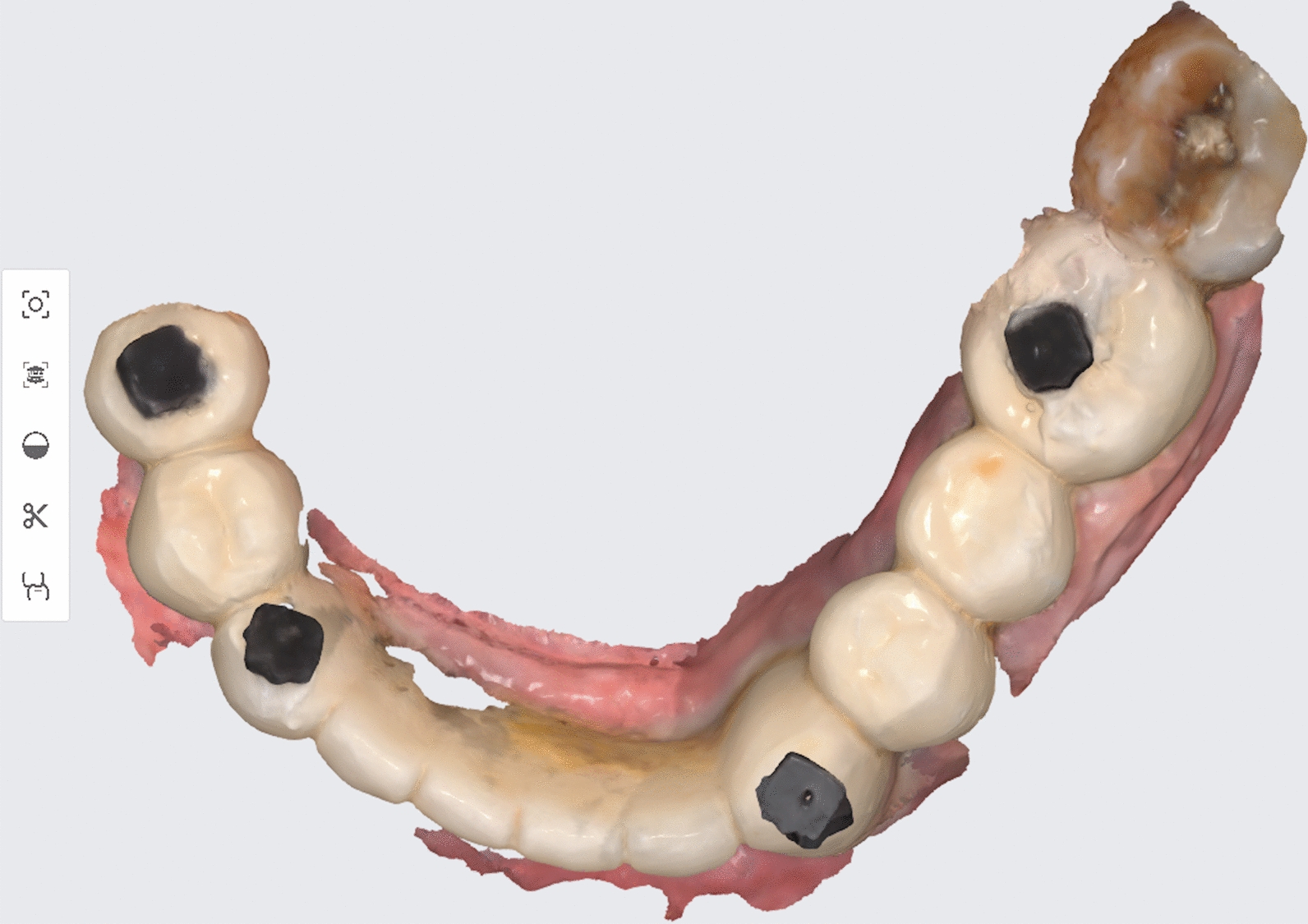
Intra-oral scanning of long-span fixed prosthesis with reference markers bonded to the prosthesis

**Fig. 4:**
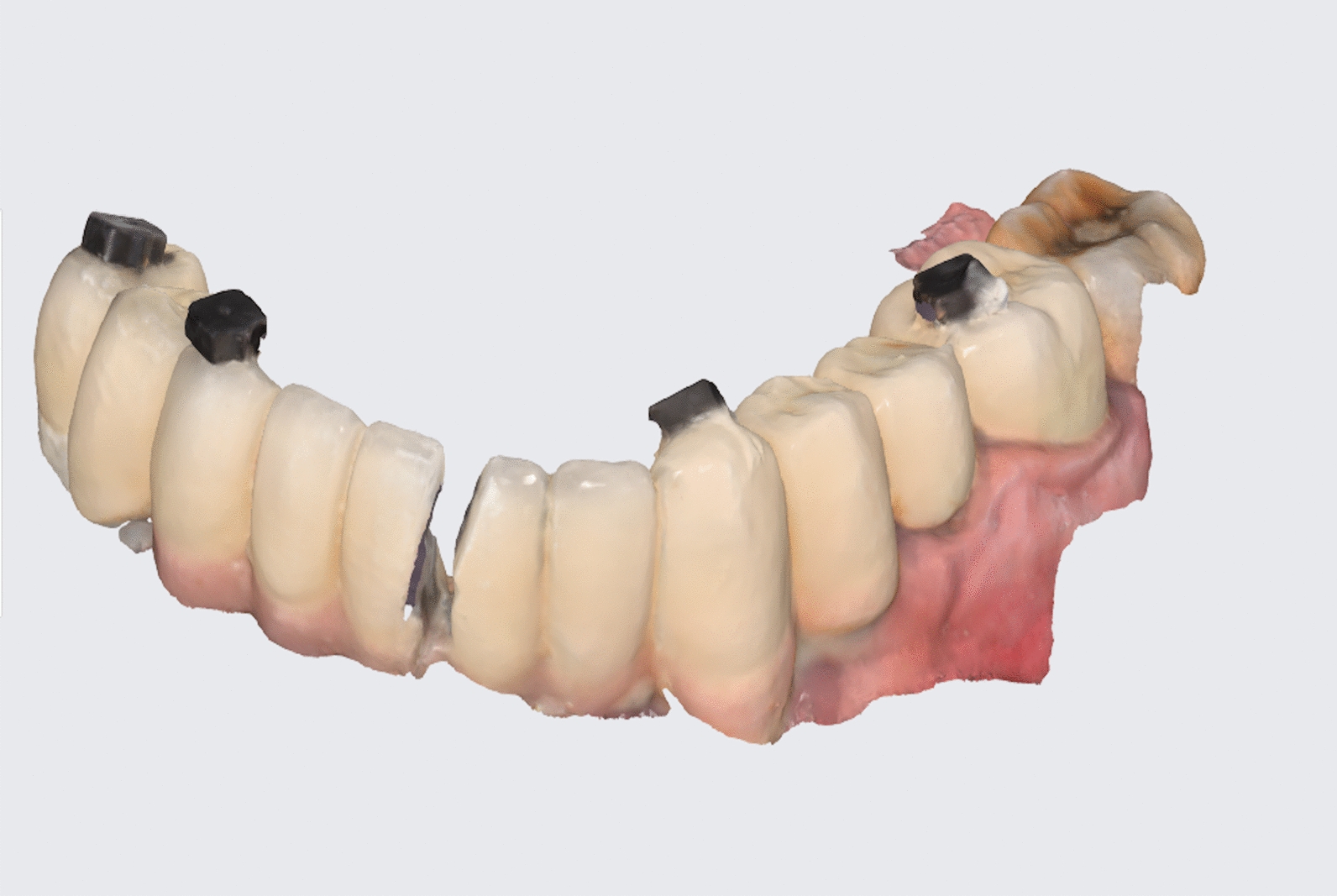
Intra-oral scanning after splitting of the wide span fixed prosthesis with reference markers bonded to the prosthesis

Three intraoral scans using (MEDIT i500; MEDIT Corp) of the prostheses were obtained. The first and second scans were made before sectioning the prosthesis at the maximal mouth opening and the minimum mouth opening sufficient for the scanner tip (18 mm), respectively [16]. The third scan was made after sectioning the prosthesis at the midline at the maximal mouth opening (Figs. 3, 4). The scan data were exported in the form of a standard tessellation language file (STL).

**Fig. 5:**
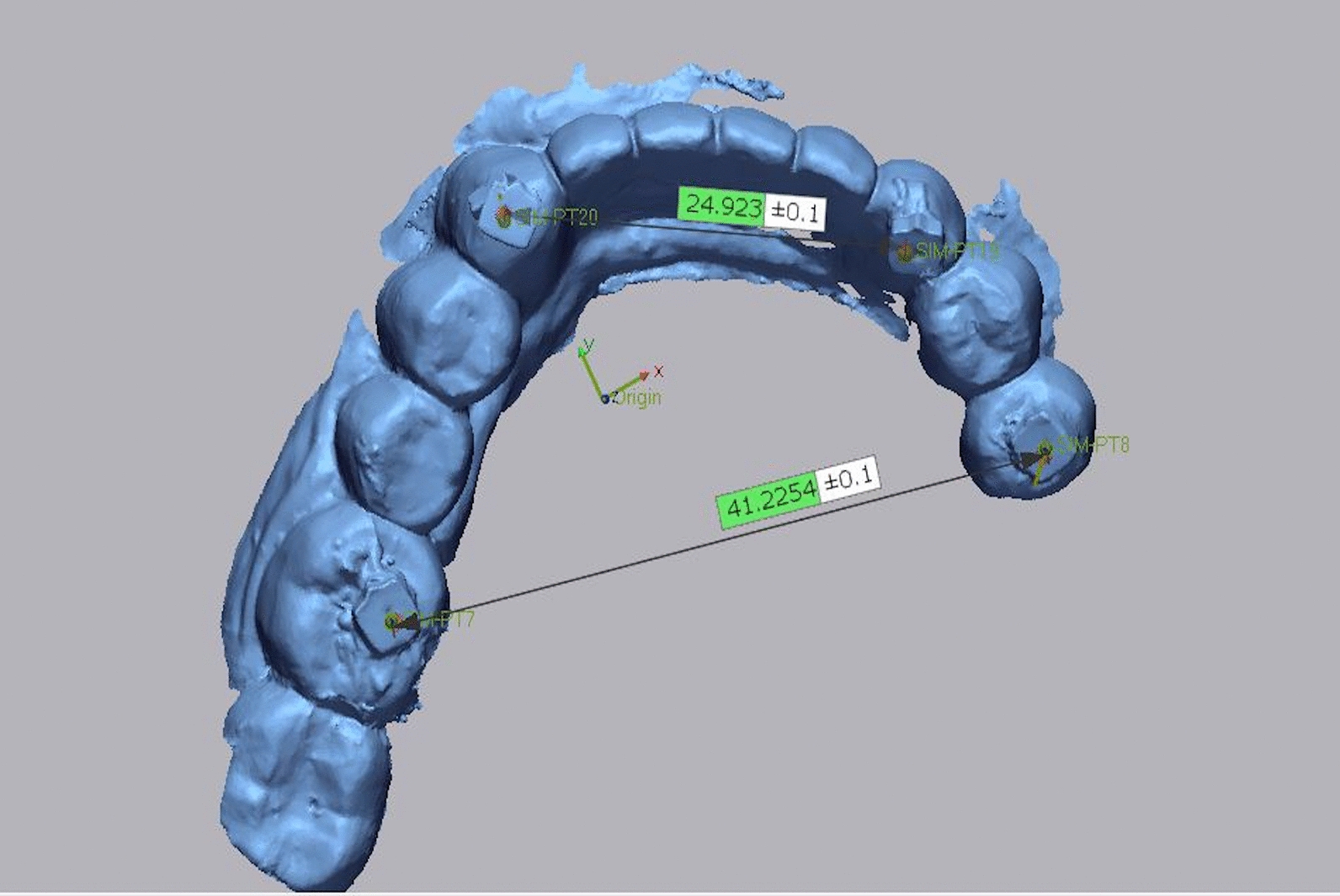
Distance measurement in mm between the reference markers in long- span fixed prosthesis

The resultant scan files were imported into 3D metrology and inspection software (Geomagic Control X; 3D Systems) [17, 18], and the distance was measured between the reference points extending from the left to the right sides of the prosthesis (from the center of the rubber stopper) (Fig. 5). To ensure comparable testing conditions, a single investigator (HIN) experienced in digital techniques performed all scans, and another investigator (MSA) conducted all the calculations to ensure inter-operator reproducibility.

The median mandibular flexure value was calculated by subtracting the measured distance between reference points at the maximum mouth opening from that of the distance between reference points at the minimum mouth opening before and after splitting of the full arch prosthesis at the midline.

### Statistical methodology

Data were collected and computed using the Statistical Package for Social Science (SPSS) statistical analysis programme (ver. 25). Kolmogorov–Smirnov test and the Shapiro–Wilk test of normality revealed no significance in the distribution of the variables, so the parametric statistics were adopted. The mean and standard deviation were used to describe the data. Comparisons were carried out between the two independent, normally distributed groups using an independent sample T-test.

## Results

**Fig. 6:**
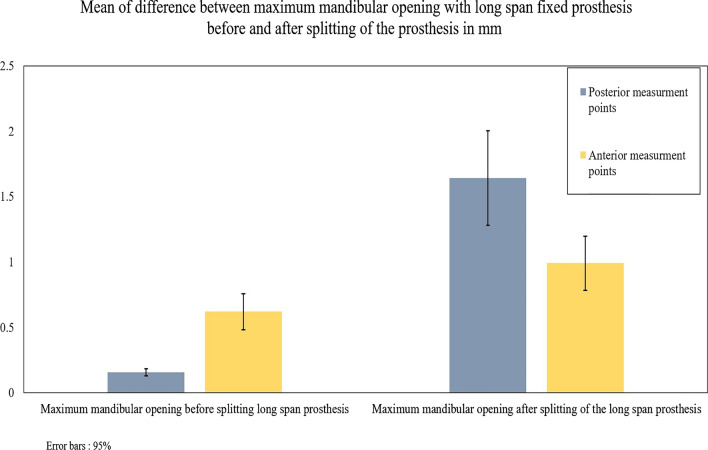
Bar chart of mean mandibular flexure before and after splitting of long-span fixed prosthesis

The results of this retrospective study showed that there was a statistically significant difference (narrowing) of the mandible (P value = 0.000) with a long-span fixed prosthesis before (0.158 ± 0.027 mm) and after sectioning (1.643 ± 0.363 mm) of the prosthesis at the midline (Table 1) (Fig. 6). It also showed less deviation in the anterior (0.992 ± 0.207 mm) than the posterior reference aids after splitting of the prosthesis.

**Table 1:** Independent sample T-test comparing difference between maximum and minimum mandibular opening with long span fixed prosthesis before and after splitting of the prosthesis

Groups	N	Difference between maximum and minimum mandibular opening with long-span fixed prosthesis	Difference between maximum and minimum mandibular opening after midline splitting of the long-span fixed prosthesis	P. value
		All measures in mm		
Mean ± standard deviation of differences between posterior measurement points	**9**	0.158 ± 0.027	1.643 ± 0.363	0.000 *
Mean ± standard deviation of differences between anterior measurement points	**9**	0.62 ± 0.139	0.992 ± 0.207	0.000 *

Bold values indicate the number of patients

*Statistically significant (p < 0.05)

*N* number of patients

The results showed that there was no statistical difference between males and females in this study (males 1.79 ± 0.371 mm and females 1.46 ± 0.294 mm) (Table 2).

**Table 2:** Independent sample T-test comparing the amount of mandibular flexure between male and female patients having long span fixed prosthesis

Groups	Difference between maximum and minimum mandibular opening with long-span fixed prosthesis			Difference between maximum and minimum mandibular opening after midline splitting of the long-span fixed prosthesis		
	Male	Female	P. value	Male	Female	P. value
N	5	4		5	4	
Mean ± standard deviation of differences between posterior measurement points	0.155 ± 0.025	0.16 ± 0.034	0.809 NS	1.79 ± 0.371	1.46 ± 0.294	0.139 NS
Mean ± standard deviation of differences between anterior measurement points	0.66 ± 0.173	0.57 ± 0.076	0.373 NS	0.958 ± 0.234	1.036 ± 0.193	0.607 NS

*NS* statistically not significant (p ≥ 0.05)

## Discussion

This retrospective cohort study allowed analysis of the mandibular flexure that has already occurred over a very long period of time (10–15 years) of long-span fixed prosthesis usage. A strength of the retrospective study is that it can accumulate data for a group of patients over longer study periods. It is also, a strength that patients are unselected and come from the real world.

A retrospective cohort study allows the investigator to describe what happens in a population of patients over time or obtain basic measures of association and correlation that will help clinicians and researchers develop future studies and interventions. The exposure and outcome information in a cohort study is collected retrospectively by using administrative datasets, reviewing patient charts, conducting history interviews, obtaining radiographs, etc. One of the weaknesses of retrospective studies is that they consider data that was common in areas the authors believed were important [19].

There are controversial debates about the flexure of the mandible during the wide opening of the mouth. This discussion was evoked for decades. Moreover, this discussion continues to guide dental education and the daily practice of dentists and dental technicians in laboratories, especially when it comes to long-span restorations.

While some authors reported such a significant difference in the width of the mandible between maximum opening and limited mouth opening, others indicated that the differences were insignificant clinically due to the effect of the periodontal ligament, which is considered the main factor involved in preventing an increase of stress/strain and bone loss around teeth due to mandibular flexure during functional movements or other types of movements of the mandible. However, some of the conclusions drawn in both groups were the result of the methods used to determine these deformations, with all their inherent limitations at the time [20–22].

However, most of the available data relies on studies that were conducted long ago [3, 10, 23, 24], which showed an extremely high variation in the magnitude of the deformation observed. Goodkind and Herinlake [6] indicated deviations ranging from 32 to 77 μm, whereas McDowell and Regli [23] reported deviations up to 1400 μm. It should be noted that, in many cases, the data obtained was collected using simple measuring devices and techniques available at that time.

Most publications compare the models made from conventional impressions that were made at minimum and maximum mouth opening [3, 5, 25–27]. These conventional methods are not accurate as digital techniques [26]. Because impression-making and model fabrication could have possible errors, also it is difficult to control the degree of mouth opening during conventional impression procedure. Nowadays. The digital techniques has been proved to be a precise and reliable method for measurement of accuracy [27]. In this study, a modification of the technique developed by Shmidt et al. [10] to measure mandibular flexure was adopted by using a black endodontic rubber stopper as a reference aid, to be clearly readable by the intraoral acquisition system. The results of this study indicated that there was a significant difference (narrowing) between the reference aid distances that were placed on the posterior region before and after splitting the wide-span rigid fixed prosthesis. The splitting of the rigid prosthesis at the midline allowed the influence of mandibular flexure stresses to be evident in the form of these deviations and the narrowing of the width of the mandible. The degree of deviation reported in this study (1.643 ± 0.363 mm) was within ranges reported in previous studies (from a few micrometers to around 1 mm). In addition, the deviation values in the anterior measuring reference aid (canines) were less than those recorded in the posterior reference aids (molars). The results of this study showed no statistical difference in mandibular deviations between males and females, despite those other reports indicating more flexure in females [28–30].

Mandibular flexure has been reported to induce negative effects in natural teeth and implant abutments after using rigid and long-span fixed prostheses [3, 25–27, 31]. In this study, the severe bone resorption around the abutment supporting the long-span fixed prosthesis was evident in all patients. This may be because this prosthesis served for a very long time, which allowed these problems to accumulate and become accentuated [16]. In this study, all patients showed severe bone resorption around the canine and premolar at the corner of the mouth, where the flexure stresses of the rigid connection of the fixed prosthesis may be most manifested [7].

The direct intraoral scanning used in this study eliminated errors that may result from distortion of the impression material and investment materials with their inherent dimensional changes [32]. The dimensional accuracy of digital models obtained by intraoral scanning was found to be higher compared to models obtained by scanning conventional impressions. [33, 34] The intraoral scanner used in this study (Medit i500) has high resolution and vivid color, which allowed the investigators to locate reference aids as well as the distinction between tooth structure and soft tissue.

The treatment plan formulated in this study included the removal of the long-span fixed prosthesis and the involved teeth and managing the cystic lesions and severe bone resorption observed around these teeth. Finally, the patients were restored with non-splinted (segmented) implant-supported prostheses.

Mandibular flexure is a multifactorial phenomenon that incorporates many variables; therefore, this study had some limitations, such as a small sample size and a short follow-up time. Another limitation of the study is that possible deformations of the mandible during mouth opening, but not during forwarding movements of the mandible, were recorded. Also, variations in periodontal ligament thickness in different patients may affect the outcome of this prosthesis. In addition, in this study, the measurement of mandibular flexure was carried out horizontally in two directions (buccal and lingual) only, whereas it naturally occurs in three directions [16]. Also, the difference in muscle strength and activity between the participants is very difficult to evaluate. Another limitation is that this study can only be conducted on dentate patients, but it is difficult to conduct it on partially or completely edentulous patients.

The sample size was nine patients because, in a retrospective study, only patients who suffered from the consequences of a long-span prosthesis supported by natural teeth were allocated for treatment in the past 2 years. Further longitudinal studies that evaluate all the factors influencing the mandibular flexure phenomenon are recommended.

This study recommends that the design of a long-span fixed prosthesis should be segmented, if possible, to allow mandibular flexure, as indicated by many previous studies [35, 36].

## Conclusion

Digital analysis of the data collected from the patients in this retrospective study indicated that deviations occur during mandibular flexure that could affect the long-term success of a single full-arch mandibular fixed prosthesis.

## Data Availability

All data generated or analyzed during the current study are included in this published article and its additional files.

## References

[1] Fischman B (1990). The rotational aspect of mandibular flexure. J Prosthet Dent.

[2] Varvara G, Feragalli B, Turkyilmaz I, D’Alonzo A, Rinaldi F, Bianchi S (2022). Prevalence and characteristics of accessory mandibular canals: a cone-beam computed tomography study in a European adult population. Diagnostics.

[3] Regli CP, Kelly EK (1967). The phenomenon of decreased mandibular arch width in opening movements. J Prosthet Dent.

[4] De Marco TJ, Paine S (1974). Mandibular dimensional change. J Prosthet Dent.

[5] Omar R, Wise MD (1981). Mandibular flexure associated with muscle force applied in the retruded axis position. J Oral Rehabil.

[6] Goodkind RJ, Heringlake CB (1973). Mandibular flexure in opening and closing movements. J Prosthet Dent.

[7] Abadzhiev M, Todorov GKK (2017). Mandibular flexure—a reason for chronic pain syndrome in edentulous patient restored with fixed ZrO_2_ construction over implants, inserted in natural bone and bone graft area. Case report. J IMAB.

[8] El-Sheikh AM, Abdel-Latif HH, Howell PGTHJ, El-Sheikh AM, Abdel-Latif HH, Howell PG, Hobkirk JA (2007). Midline mandibular deformation during non-masticatory functional movements in edentulous subjects with dental implants. Int J Oral Maxillofac Implant.

[9] Nokar SNR (2005). The effect of superstructure design on stress analysis. Int J Oral Maxillofac Implant.

[10] Schmidt A, Klussmann L, Schlenz MA, Wöstmann B (2021). Elastic deformation of the mandibular jaw revisited—a clinical comparison between digital and conventional impressions using a reference. Clin Oral Investig.

[11] Zarone F, Apicella A, Nicolais L, Aversa RSR, Zarone F, Apicella A, Nicolais L, Aversa R, Sorrentino R (2003). Mandibular flexure and stress build-up in mandibular full-arch fixed prostheses supported by osseointegrated implants. Clin Oral Implant Res.

[12] Law C, Bennani V, Lyons K, Swain M (2012). Mandibular flexure and its significance on implant fixed prostheses: a review. J Prosthodont.

[13] Bianchi S, Bernardi S, Mattei A, Cristiano L, Mancini L, Torge D (2022). Morphological and biological evaluations of human periodontal ligament fibroblasts in contact with different bovine bone grafts treated with low-temperature deproteinisation protocol. Int J Mol Sci.

[14] Bianchi S, Bernardi S, Simeone D, Torge D, Macchiarelli G, Marchetti E (2022). Proliferation and morphological assessment of human periodontal ligament fibroblast towards bovine pericardium membranes: an in vitro study. Materials.

[15] Cao YT, Gu QH, Wang YW, Jiang Q (2022). Enucleation combined with guided bone regeneration in small and medium-sized odontogenic jaw cysts. World J Clin Cases.

[16] Gülsoy M, Tuna SH, Pekkan G (2022). Evaluation of median mandibular flexure values in dentulous and edentulous subjects by using an intraoral digital scanner. J Adv Prosthodont.

[17] Dohiem MM, Abdelaziz MS, Abdalla MF, Fawzy AM (2022). Digital assessment of the accuracy of implant impression techniques in free end saddle partially edentulous patients. A controlled clinical trial. BMC Oral Health.

[18] Dohiem MM, Emam NS, Abdallah MF, Abdelaziz MS (2022). Accuracy of digital auricular impression using intraoral scanner versus conventional impression technique for ear rehabilitation: a controlled clinical trial. J Plast Reconstr Aesthet Surg.

[19] Talari K, Goyal M (2020). Retrospective studies—utility and caveats. J R Coll Physicians Edinb.

[20] Horiuchi M, Ichikawa T, Noda M, Matsumoto N (1997). Use of interimplant displacement to measure mandibular distortion during jaw movements in humans. Arch Oral Biol.

[21] Korioth TWP, Hannam AG (1994). Deformation of the human mandible during simulated tooth clenching. J Dent Res.

[22] Sivaraman K, Chopra A, Venkatesh SB (2016). Clinical importance of median mandibular flexure in oral rehabilitation: a review. J Oral Rehabil.

[23] McDowell JA, Regli CP (1961). A quantitative analysis of the decrease in width of the mandibular arch during forced movements of the mandible. J Dent Res.

[24] Burch JG, Borchers G (1970). Method for study of mandibular arch width change. J Dent Res.

[25] Gates GN, Nicholls JI (1981). Evaluation of mandibular arch width change. J Prosthet Dent.

[26] Shinkai RSA, Canabarro SDA, Schmidt CB, Sartori EA (2004). Reliability of a digital image method for measuring medial mandibular flexure in dentate subjects. J Appl Oral Sci.

[27] Keul C, Güth JF (2020). Accuracy of full-arch digital impressions: an in vitro and in vivo comparison. Clin Oral Investig.

[28] Ebadian B, Abolhasani M, Heidarpour A, Ziaei M, Jowkar M (2020). Assessment of the relationship between maximum occlusal force and median mandibular flexure in adults: a clinical trial study. J Indian Prosthodont Soc.

[29] Wolf L, Bergauer B, Adler W, Wichmann M, Matta RE (2019). Three-dimensional evaluation of mandibular deformation during mouth opening. Int J Comput Dent.

[30] Choi AH, Conway RC, Taraschi V, Ben-Nissan B (2015). Biomechanics and functional distortion of the human mandible. J Investig Clin Dent.

[31] Abdelaziz MS, Fawzy A, Ghali RMNH (2022). Retention loss of locator attachment system different retention caps for two implant retained mandibular overdenture. Futur Dent J.

[32] Quaas S, Rudolph H, Luthardt RG (2007). Direct mechanical data acquisition of dental impressions for the manufacturing of CAD/CAM restorations. J Dent.

[33] Flügge TV, Schlager K, Nelson SNM (2013). Precision of intraoral digital dental impressions with iTero and extraoral digitization with the iTero and a model scanner. Am J Orthod Dentofac Orthop.

[34] Anh JW, Park JM, Chun YS, Kim M, Kim M (2016). A comparison of the precision of three-dimensional images acquired by 2 digital intraoral scanners: effects of tooth irregularity and scanning direction. Korean J Orthod.

[35] Gao J, Li X, He J, Jiang L, Zhao B (2022). The effect of mandibular flexure on the design of implant-supported fixed restorations of different facial types under two loading conditions by three-dimensional finite element analysis. Bioeng Biotechnol.

[36] Ahmed MHM, Diederich H, Abo Heikal MM (2018). Consequence of midline mandibular flexure on bilaterally splinted and non-splinted implant-supported mandibular full arch prosthesis with immediately loaded implants: a one year clinical study. Mouth Teeth.

